# What we say to families of intensive care unit patients - do they understand?

**DOI:** 10.1186/2197-425X-3-S1-A471

**Published:** 2015-10-01

**Authors:** S Barbosa, A Ilchenko, A Carmo, F Fernandes, S Nascimento, C Granja

**Affiliations:** Centro Hospitalar do Algarve, Hospital de Faro, Emergency and Intensive Care Department, Faro, Portugal; University of Algarve, Department of Biomedical Sciences and Medicine, Faro, Portugal; Faculty of Medicine of Porto, CINTESIS, Porto, Portugal

## Introduction

Effective communication between physicians, nurses and the patient’s representatives in intensive care unit (ICU) is a vital component of quality of care.

## Objectives

To evaluate the communication between ICU patient’s representatives and caregivers and identify factors associated with poor comprehension using a protocol previously described [[1]].

## Methods

Prospective observational study running from 15 October 2014 to 15 March 2015. Included all patients who stayed for more than 2 days and had at least one visit in the first 5 days of ICU stay. The investigators applied three questionnaires: the representative (a relative or a friend that was designated by the patient or by the family and that was present in the first physician-family meeting); the physician that met the family at the admission; the nurse who cared the patient for more than 2 days.

## Results

Of the 207 patients admitted in this period, 135 stayed more than 2 days in the ICU. Of these, we were able to enroll the family in 63 of them (47%). Forty-five patients (71%) had more than 50 years, 52% were male, 71% were married, 84% were Portuguese and 78% had comorbidities. Median SAPS II was 47 and median ICU length of stay was 11 days. Main reasons for ICU admission were shock (35%), respiratory infection with acute respiratory failure (26%) and multiple trauma (13%). Thirty-eight of the representatives (60%) were female, 86% were Portuguese, 38% were sons, 76% were employed and 24% had only primary school education. The first physician-family meeting duration was between 10 and 20 minutes in 62% and occurred in a room used only for this purpose in 43%. Failure to comprehend the diagnosis, prognosis or treatment was noted in 39 representatives (62%); 25% of the representatives did not understand the diagnosis, 49% did not understand the treatment and 37% did not understand the prognosis. Factors significantly associated with poor comprehension were patient comorbidities, age of the representative more than 50 years and first physician-family meeting duration less than 10 minutes. Also, lower number of daily visits and Portuguese nationality, although not reaching statistical significance, exhibited a trend to be associated with poor comprehension.

## Conclusions

In this study poor comprehension was found in 62% of the representatives, which is in line with previous studies [[1]]. A high number of comorbidities, first physician-family meeting duration less than 10min, representatives older than 50 years, were the factors significantly associated to poor comprehension. This study draws our attention to the need to improve communication with the families and to try to identify patients and families at high risk of poor comprehension.
